# Predictor of outcome after living donor liver transplantation for patients with hepatocellular carcinoma beyond the Japan criteria

**DOI:** 10.1002/ags3.12335

**Published:** 2020-04-24

**Authors:** Yusuke Yonemura, Tomoharu Yoshizumi, Shoichi Inokuchi, Yukiko Kosai‐Fujimoto, Noboru Harada, Shinji Itoh, Takeo Toshima, Kazuki Takeishi, Shohei Yoshiya, Masaki Mori

**Affiliations:** ^1^ Department of Surgery Oita Prefectural Hospital Oita Japan; ^2^ Department of Surgery and Science Graduate School of Medical Sciences Kyushu University Fukuoka Japan

**Keywords:** alpha‐fetoprotein, des‐gamma‐carboxy prothrombin, hepatocellular carcinoma, Japan criteria, living donor liver transplantation

## Abstract

**Background:**

The Japan criteria (JC, maximum tumor size within 5 cm, within five tumor nodules, AFP within 500 ng/mL or within Milan criteria) have been applied to cadaveric liver transplantation (LT) for hepatocellular carcinoma (HCC) and will be used for living donor LT (LDLT) in Japan. The aim of this study was to verify the JC in LDLT and to clarify the risk factor of HCC recurrence and mortality after LDLT beyond the JC.

**Patients and methods:**

Adult patients who underwent LDLT for end‐stage liver disease with HCC until October 2019 were reviewed retrospectively (n = 246). Patients were divided into two groups according to whether they were within JC (n = 203) or beyond JC (n = 43). Recurrence‐free or overall survival rates after LDLT were compared. Univariate and multivariate analyses were performed to identify risk factors of HCC recurrence and HCC‐related mortality after LDLT for patients beyond the JC.

**Results:**

Patients beyond the JC had significantly poorer 5‐year recurrence‐free (50.3% vs 95.9%, *P* < .001) or overall (61.7% vs 98.1%, *P* < .001) survival rates compared with patients within the JC. A multivariate analysis revealed that des‐gamma‐carboxy prothrombin (DCP) ≥ 300 mAU/mL (hazard ratio 9.36, 95% CI; 2.41‐36.4, *P* = .001) was an independent risk factor for HCC recurrence and HCC‐related mortality (hazard ratio 13.8, 95% CI; 1.92‐98.6, *P* = .01) after LDLT in patients beyond the JC.

**Conclusion:**

The outcome of LDLT for patients within the JC was favorable. Patients beyond the JC with DCP ≥ 300 mAU/mL might be contraindicated for LDLT.

## Graphical abstract

The aim of this study was to clarify the risk factor of HCC recurrence and mortality after LDLT beyond the Japan criteria (JC). We revealed that des‐gamma‐carboxy prothrombin (DCP) ≥300 mAU/ml was an independent risk factor for tumor recurrence and recipient HCC‐related mortality after LDLT in patients beyond the JC.

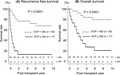

**Abbreviations**
AFP: alpha‐fetoprotein
CT: computed tomography
DCP: des‐gamma‐carboxy prothrombin
GRWR: graft recipient weight ratio
GW: graft weight
HCC: hepatocellular carcinoma
HCV: hepatitis C virus
JC: Japan criteria
LDLT: living donor liver transplantation
LMR: lymphocyte‐to‐monocyte ratio
LT: liver transplantation
MC: Milan criteria
MELD: model for end‐stage liver disease
MMF: mycophenolate mofetil
NLR: neutrophil‐lymphocyte ratio
SLW: standard liver weight
TAM: tumor‐associated macrophage

## 1 INTRODUCTION

Hepatocellular carcinoma (HCC) is the most frequent primary liver cancer and the third most common cause of cancer‐related death.^1^ Its incidence is increasing worldwide because of the dissemination of hepatitis virus infection and increase of alcoholic or non‐alcoholic steatohepatitis. Liver transplantation (LT), which offers the theoretical advantage of removing both the tumor and the organ that are at risk of developing future malignancy, is an established therapy for HCC in patients with liver cirrhosis.^2^, ^3^ In Asian countries, religious, cultural, and political ideologies have created significant obstacles to the transplantation of cadaver organs. Organ shortages have forced patients with HCC to endure long waiting periods that are associated with tumor development. Thus, living donor LT (LDLT) is a choice for treating such HCC patients after treatments including radio frequency ablation, transarterial chemoembolization, and/or hepatic resection.^4^ It is important to allocate the deceased donor livers to ensure a reasonable outcome for living donors who need to undergo invasive surgery.^3^

The Milan criteria (MC) have significantly improved the outcome of LT for HCC and have become the gold standard to achieve a favorable outcome after LT for HCC.^5^ Because Japanese national insurance has covered LDLT for HCC within the MC since 2004, the number of LDLT patients within the MC is increasing in Japan. LT for patients within the MC generally reach a 5‐year overall survival rate of 70%‐80% and a recurrence rate of around 10%. Many groups have proposed LT for patients with large and numerous tumors; however, these favorable outcomes have raised the question of whether the selection criteria might be expanded.^6^, ^7^ Another criticism against the MC is the lack of tumor‐related biological indices to help dictate best oncological practice when transplanting HCC patients. Therefore, several centers have developed criteria that include tumor biological indices such as alpha‐fetoprotein (AFP) or des‐gamma‐carboxy prothrombin (DCP) indices to predict outcomes.^8^, ^9^ The Japanese Liver Transplantation Society recently proposed expanded criteria based on a retrospective data analysis of a Japanese nationwide survey.^10^ These criteria are termed the 5‐5‐500 rule and are as follows: maximum tumor size within 5 cm, within five tumor nodules, and AFP within 500 ng/mL. The Japan criteria (JC), which include the 5‐5‐500 rule or within Milan criteria, have been applied to selection criteria for cadaveric LT for HCC and will be used for LDLT very soon in Japan. Patients beyond the JC will not be covered by national insurance, but they will have a chance to undergo LDLT at personal cost. Therefore, it is important to reveal the contraindication of LDLT for such patients.

The aim of this study was to verify the Japan criteria in LDLT and to clarify the risk factor of HCC recurrence and mortality after LDLT beyond the Japan criteria to reveal the contraindication of LDLT in patients beyond the Japan criteria.

## 2 PATIENTS AND METHODS

### 2.1 Patients

The study protocol was carried out in accordance with The Code of Ethics of the World Medical Association (Declaration of Helsinki) and the institutional review board (Approved number 2019‐186). Two hundred and forty‐six adult patients underwent LDLT for end‐stage liver disease with HCC at Kyushu University Hospital between April 1999 and October 2019. A pre‐transplant imaging study revealed that 203 patients were within the JC and 43 were beyond the JC. Among 203 patients within the JC, 26 recipients underwent LDLT for indications other than HCC and were included in this study because HCC was found upon explant pathology. One hundred and fifty‐seven patients underwent pre‐transplant treatment for HCC including radio frequency ablation, transarterial chemoembolization, microwave coagulation therapy, and/or hepatic resection depending on the patient's liver function and tumor status. Graft types included left lobe with caudate lobe (LL+C) graft (n = 130), right lobe graft without the middle hepatic vein (n = 109), posterior segment graft (n = 6), and dual graft (n = 1). The etiology of liver cirrhosis was hepatitis C (n = 165), hepatitis B (n = 40), and others (n = 41). Our selection criteria to perform LDLT for HCC patients were as follows: (a) no modality except LDLT available to cure patients with HCC; (b) no extra‐hepatic metastasis; and (c) no major vascular infiltration. There were no restrictions on tumor size, number of nodules, or pre‐transplant treatment. Because we proposed our own criteria, we did not perform LDLT for HCC patients who had both tumor size > 5 cm and DCP level > 300 mAU/mL.^11^

Pre‐transplant imaging was used to estimate the maximum tumor size and number of nodules. AFP, DCP, neutrophil‐to‐lymphocyte ratio (NLR), and lymphocyte‐to‐monocyte ratio (LMR) were measured just prior to LDLT.^12^ The histological grades obtained from the explanted livers were used for tumor differentiation and the presence of vascular invasion.

### 2.2 Donor and graft selection

Donors were selected from candidates who hoped to be living donors.^13^ Donors were required to be within the third degree of consanguinity with recipients or spouses, and to be between 20 and 65 years of age. For a donor who was not within the third degree of consanguinity, individual approval was obtained from the Ethics Committee of Kyushu University Hospital. Good Samaritan donations were not used.

Eligible donors proceeded to the imaging studies, including chest and abdominal X‐rays and 1‐mm‐slice computed tomography (CT) scans for graft volumetric analysis. Three‐dimensional CT was introduced for the volumetric analysis and delineation of vascular anatomy. The standard liver weight (SLW) of recipients was calculated according to the formula of Urata.^14^ Graft weight (GW) was predicted by CT volumetric analysis. Our usual decision about graft type for recipients was based on the preoperatively predicted GW to SLW ratio.^15^ LL + C graft was used when the preoperatively predicted GW–SLW ratio was ≥35%. When GW–SLW ratio with LL + C graft was <35% and remnant donor liver volume after right lobectomy was ≥35%, right lobe graft was used. Posterior segment graft was considered when the donor's vascular anatomy was suitable to take a posterior segment.^13^

### 2.3 Postoperative management

The graft retrieval technique, recipient surgery, and perioperative management of the recipients, including immunosuppression regimens, were described previously.^4^, ^11^ Splenectomy was performed when patients had preoperative low predicted GW–SLW ratio (35% or less), portal hypertension indicated by a large portosystemic shunt, splenomegaly, risky esophagogastric varices, high portal pressure (>20 mm Hg) after unclamping, ABO blood type‐incompatible donor, positive HCV RNA, or severe hypersplenism defined as a preoperative WBC count < 1000/mL and/or platelet count < 50 000/mL.^16^ Immunosuppression was initiated using a protocol based on either tacrolimus (Prograf; Astellas Pharma Inc.) or cyclosporine A (Neoral; Novartis Pharma KK) with steroid and/or mycophenolate mofetil (MMF; Chugai Pharmaceutical Co. Ltd.). A target trough level of tacrolimus was set at 10 ng/mL for 3 months after LDLT, followed by 5‐10 ng/mL thereafter. A target trough level of cyclosporine A was set at 250 ng/mL for 3 months after LDLT, followed by 150‐200 ng/mL thereafter. Methylprednisolone was initiated on the day of LDLT, tapered and converted to prednisolone 7 days after LDLT. Prednisolone treatment was tapered and discontinued 6 months after LDLT. MMF was started at 1‐2 g/d on the day after LDLT, tapered and discontinued until 6 months after LDLT. A trough level was not measured for MMF. Everolimus was covered by national insurance in 2018 and was used 1‐3 months after LDLT for cases beyond the MC.^17^

All patients had monthly follow‐ups, and the median follow‐up period was 2595 days, with 983 days and 4363 days as the 25th and 75th percentiles, respectively. Tumor markers were routinely checked every 3 months. Chest/abdominal CT scan and bone scintigraphy were routinely performed every 6 months after LDLT to rule out HCC recurrence.

### 2.4 Post‐LDLT HCC recurrence and recipient HCC‐related mortality

Hepatocellular carcinoma recurrence after LDLT was set as the primary end point. HCC recurrence was defined when any imaging studies, such as chest or abdominal CT scan, or bone scintigraphy revealed the recurrence of HCC. Recurrence‐free survival was defined as the period between LDLT and tumor recurrence. Patient HCC‐related mortality after the LDLT was set as the secondary end point. Overall survival was defined as the time period between LDLT and recipient HCC‐related death.

### 2.5 Statistical analysis

Recurrence‐free or overall survival rates were calculated by the Kaplan–Meier product‐limited method. Data were expressed as means. All statistical analyses were performed using JMP 14.0 software (SAS, Inc.). A *P*‐value of < 0.05 was considered significant. Optimal cut‐off values of NLR (> 2.2), LMR (> 2.75), DCP (≥ 300), number of tumor nodules (≥25), or model for end‐stage liver disease (MELD > 20) for the prediction of HCC recurrence after LDLT were set using receiver operating characteristics (ROC) curves. Although the ROC curve indicated that cut‐off values were 3.5 cm for tumor size and 100 ng/mL for AFP, the cut‐off value for tumor size was set at 5.0 cm and the value for AFP was set at 500 because JC includes a maximum tumor size within 5 cm and AFP within 500 ng/mL. These cut‐off values were used for the prediction of recipient HCC‐related mortality. Variables that had a *P*‐value less than .2 by univariate analyses were used for multivariate analyses.

## 3 RESULTS

The characteristics of the patients and donors are shown in Table 1. Beyond the JC group included younger, more hepatitis C virus (HCV) positive and diabetic patients, patients with lower MELD score, patients requiring longer surgery time, patients with lower frequency of simultaneous splenectomy, and patients with transplanted grafts from younger donors. More LL+C or posterior grafts were used in recipients beyond the JC; thus, GRWR was smaller in patients beyond the JC.

**TABLE 1:** Characteristics of patients and donors

Variables		Within the JC (n = 203)	Beyond the JC (n = 43)	*P*‐value
Recipient	Gender (Male, %)	55.7	67.4	.15
	Age (years)	59.1	56.5	.04
	HCV (%)	64.5	81.4	.03
	HBV (%)	17.7	9.3	.15
	MELD score	13.6	11.6	.03
	Diabetes Mellitus (Yes, %)	20.2	34.9	.045
	Surgery time (min)	766	831	.02
	Blood loss (mL)	7169	7340	.95
	Splenectomy (Yes, %)	72.9	55.8	.03
Donor	Gender (Male, %)	69.0	65.1	.94
	Age (years, range)	36.4	30.6	.001
	Graft (Right lobe, %)	47.8	27.9	.008
	GW‐SLW ratio (%)	41.4	39.3	.13
	GRWR (%)	0.78	0.72	.018
	ABO incompatible (%)	15.8	4.7	.08
Tumor	Maximum size (cm)^a^	1.9	3.3	<.0001
	Number of nodules (median)^a^	1	8	<.0001
	Bilobar distribution (yes, %)	26.1	86.1	<.0001
	Milan criteria (yes, %)	84.7	0	<.0001
	Pre‐transplant NLR	3.50	3.25	.81
	Pre‐transplant LMR	3.43	2.87	.09
	Pre‐transplant AFP (ng/mL)	268	3141	<.0001
	Pre‐transplant DCP (mAU/mL)	234	1081	.0002
	Pre‐LDLT treatment (yes, %)	59.6	83.7	. 002
	Times of Pre‐LDLT Treatment (median)	1	3	.0008
	Maximum size on pathology (cm)	2.0	3.6	<.0001
	Number of nodules on pathology (median)^a^	2	18	<.0001
	Micro vascular invasion (Yes, %)	17.2	65.1	<.0001
	Bilobar distribution on pathology (yes, %)	36.0	88.4	<.0001
	Within JC by explant pathology (yes, %)	82.3	2.3	<.0001
	Pathological poorly differentiation	14.8	53.5	<.0001

Abbreviations: AFP, alpha‐fetoprotein; DCP, des‐gamma‐carboxy prothrombin; ELD, model for end‐stage liver disease; GRWR, graft recipient weight ratio; MR, lymphocyte‐to‐monocyte ratio; NLR, neutrophil‐to‐lymphocyte ratio; SLW, standard liver weight; W, graft weight.

^a^ Twenty‐six cases underwent LDLT for indications other than HCC and were included in this study because HCC was found upon explant pathology.

Tumors were distributed in bilateral lobes in patients beyond the JC. Pre‐transplant AFP or DCP was higher in patients beyond the JC, whereas pre‐transplant NLR or LMR was not different between the two groups. More pre‐transplant treatment for HCC was performed in patients beyond the JC. Explant pathology revealed greater microvascular invasion and poor pathological differentiation in patients beyond the JC. Pathological examination revealed 17.7% patients within the JC by pre‐transplant imaging were beyond the JC. In contrast, 2.3% were within the JC in patients who at pre‐transplant were beyond the JC.

**FIGURE 1:**
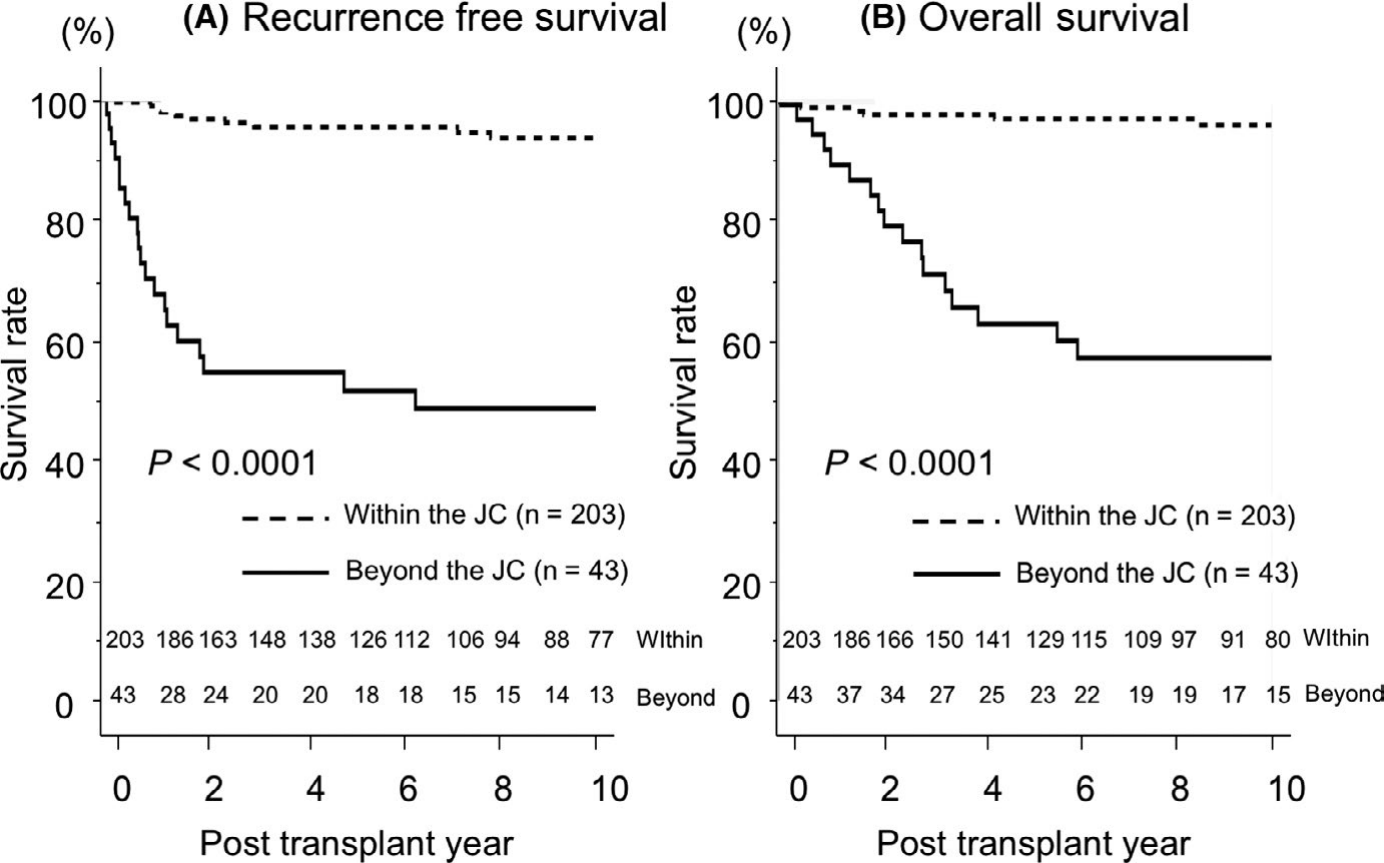
Recurrence‐free and overall survival after LDLT for HCC. A, Recurrence‐free survival of recipients divided according to within (n = 203) or beyond (n = 43) the Japan criteria (JC). B, Overall survival of recipients divided according to within (n = 203) or beyond (n = 43) the JC

The 1‐, 5‐, and 10‐year recurrence‐free survival rates in patients within the JC were 100%, 95.9%, and 94.0%, respectively. In contrast, the rates in patients beyond the JC were 68.4%, 50.3%, and 47.3%, respectively (*P* < .0001, Figure 1A). The 1‐, 5‐, and 10‐year overall survival rates in patients within the JC were 100%, 98.1%, and 97.1%, respectively. In contrast, the rates in patients beyond the JC were 90.2%, 61.7%, and 56.1%, respectively (*P* < .0001, Figure 1B).

Among 43 patients beyond the JC, univariate analysis revealed the following risk factors for HCC recurrence: NLR > 2.2 (*P* = .03), LMR ≤ 2.75 (*P* = .01), DCP ≥ 300 mAU/mL (*P* < .0001), tumor nodules ≥ 25 (*P* = .046), and tumor size > 5 cm (*P* = .01). A multivariate analysis revealed that DCP ≥ 300 mAU/mL (hazard ratio 9.36, 95% CI; 2.41‐36.4, *P* = .001) was an independent risk factor for HCC recurrence after LDLT in patients beyond the JC (Table 2).

**TABLE 2:** Risk factors for HCC recurrence after LDLT in patients beyond the JC

Variables	Univariate analysis			Multivariate analysis		
	Hazard ratio	95% CI	*P*‐value	Hazard ratio	95% CI	*P*‐value
Pre‐LDLT NLR > 2.2: Yes vs No	3.03	1.10‐8.35	.03	3.08	0.88‐10.9	.08
Pre‐LDLT LMR > 2.75: No vs Yes	3.74	1.36‐10.3	.01	1.28	0.36‐4.51	.70
DCP ≥ 300 (mAU/mL): Yes vs No	6.19	2.50‐15.3	<.0001	9.36	2.41‐36.4	.001
Tumor nodules ≥ 25: Yes vs No	2.81	1.02‐7.74	.046	0.89	0.17‐4.59	.88
Tumor size > 5 (cm): Yes vs No	4.48	1.41‐14.2	.01	1.03	0.26‐4.04	.96
Donor male: Yes vs No	0.47	0.20‐1.12	.09	0.43	0.13‐1.48	.18
MELD > 20: Yes vs No	2.67	0.78‐9.19	.12	0.37	0.08‐1.66	.19
HCV positive: No vs Yes	1.93	0.70‐5.33	.20			
AFP > 500 (ng/mL): Yes vs No	1.70	0.72‐4.04	.23			
HBsAg positive: Yes vs No	2.07	0.61‐7.10	.25			
Pre‐LDLT treatment: Yes vs No	1.25	0.37‐4.26	.72			
Recipient male: Yes vs No	1.35	0.54‐3.36	.52			
Bilobar distribution: Yes vs No	1.85	0.43‐7.96	.41			
GW ‐ SLW ratio ≥ 35%: Yes vs No	1.04	0.38‐2.83	.95			
Splenectomy: Yes vs No	1.07	0.45‐2.56	.87			

Abbreviations: AFP, alpha‐fetoprotein; DCP, des‐gamma‐carboxy prothrombin; GW, graft weight; LDLT, living donor liver transplantation; LMR, lymphocyte‐to‐monocyte ratio; MELD, model for end‐stage liver disease; NLR, neutrophil‐to‐lymphocyte ratio; SLW, standard liver weight.

Table 3 shows the risk factors of HCC‐related mortality after LDLT among 43 patients beyond the JC. Univariate analysis revealed that NLR > 2.2 (*P* = .01), LMR ≤ 2.75 (*P* = .005), DCP ≥ 300 mAU/mL (*P* = .0002), tumor nodules ≥ 25 (*P* = .03), tumor size > 5 cm (*P* = .01) were risk factors for HCC‐related mortality. A multivariate analysis revealed that DCP ≥ 300 mAU/mL (hazard ratio 13.8, 95% CI; 1.92‐98.6, *P* = .01) and LMR ≤ 2.75 (hazard ratio 39.9, 95% CI; 1.64‐96.9, *P* = .02) were independent risk factors of patient HCC‐related mortality after LDLT in patients beyond the JC.

**TABLE 3:** Risk factors for HCC‐related mortality after LDLT in patients beyond the JC

Variables	Univariate analysis			Multivariate analysis		
	Hazard ratio	95% CI	*P*‐value	Hazard ratio	95% CI	*P*‐value
Pre‐LDLT NLR > 2.2: Yes vs No	4.71	1.35‐16.4	.01	0.32	0.01‐6.90	.47
Pre‐LDLT LMR > 2.75: No vs Yes	6.06	1.74‐21.2	.005	39.9	1.64‐96.9	.02
DCP ≥ 300 (mAU/mL): Yes vs No	7.27	2.57‐20.6	.0002	13.8	1.92‐98.6	.01
Tumor nodules ≥ 25: Yes vs No	3.12	1.10‐8.85	.03	1.17	0.15‐8.98	.88
Donor male: Yes vs No	0.44	0.17‐1.12	.08	1.45	0.28‐ 7.54	.66
Recipient male: Yes vs No	2.76	0.90‐8.44	.07	13.8	0.94‐202	.06
MELD > 20: Yes vs No	2.82	0.81‐9.86	.10	0.22	0.03‐1.67	.14
HCV positive: No vs Yes	2.04	0.73‐5.75	.18	4.77	0.42‐54.8	.21
Tumor size > 5 cm: Yes vs No	4.17	1.34‐13.0	.01	2.20	0.24‐19.9	.48
HBsAg positive: Yes vs No	2.57	0.73‐9.03	.14	2.54	0.16‐40.0	.51
AFP ≥ 500 (ng/mL): Yes vs No	1.09	0.43‐2.75	.86			
GW ‐ SLW ratio ≥ 35: No vs Yes	1.09	0.39‐3.06	.87			
Pre‐LDLT treatment: Yes vs No	0.99	0.29‐3.44	.99			
Bilobar distribution: Yes vs No	1.54	0.35‐6.69	.57			
Splenectomy: No vs Yes	1.08	0.42‐2.76	.87			

Abbreviations: AFP, alpha‐fetoprotein; DCP, des‐gamma‐carboxy prothrombin; GW, graft weight; LDLT, living donor liver transplantation; LMR, lymphocyte‐to‐monocyte ratio; MELD, model for end‐stage liver disease; NLR, neutrophil‐to‐lymphocyte ratio; SLW, standard liver weight.

Table 4 shows the characteristics of the patients and donors beyond the JC. The 43 patients were divided into two groups according to DCP < 300 mAU/mL (n = 26) and DCP ≥ 300 mAU/mL (n = 17). Patients with a DCP ≥ 300 mAU/mL had a higher MELD score, more blood loss during LDLT, bigger tumor size by imaging study and explant pathology, more tumor nodules by imaging study and explant pathology, higher AFP, higher NLR, lower LMR, more positive microvascular invasion, and bilobar distribution by pathology.

**TABLE 4:** Characteristics of patients and donors beyond the JC

Variables		DCP < 300 mAU/mL (n = 26)	DCP ≥ 300 mAU/mL (n = 17)	*P*‐value
Recipient	Gender (Male, %)	57.7	82.4	.08
	Age (years)	57.0	55.8	.53
	HCV (%)	80.8	82.4	.89
	HBV (%)	11.5	5.9	.52
	MELD score	10.0	14.0	.01
	Diabetes Mellitus (Yes, %)	34.6	35.3	.96
	Surgery time (min)	800	878	.18
	Blood loss (mL)	4388	11 855	.03
	Splenectomy (Yes, %)	57.7	52.9	.76
Donor	Gender (Male, %)	69.2	62.5	.65
	Age (years, range)	31.7	28.8	.31
	Graft (Right lobe, %)	26.9	29.4	.37
	GW‐SLW ratio (%)	39.8	38.7	.62
	GRWR (%)	0.73	0.70	.47
	ABO incompatible (%)	7.7	0.0	.15
Tumor	Maximum size (cm)	2.8	4.0	.005
	Number of nodules (median)	7	12	.01
	Bilobar distribution (yes, %)	80.8	94.1	.19
	Pre‐transplant NLR	2.19	4.86	.009
	Pre‐transplant LMR	3.29	2.23	.003
	Pre‐transplant AFP (ng/mL)	783	6747	.02
	Pre‐transplant DCP (mAU/mL)	99	2581	.0005
	Pre‐LDLT treatment (yes, %)	84.6	82.4	.84
	Times of Pre‐LDLT Treatment (median)	3	2	.52
	Maximum size on pathology (cm)	3.0	4.6	.002
	Number of nodules on pathology (median)^a^	14	33	.009
	Micro vascular invasion (Yes, %)	53.9	82.4	.049
	Bilobar distribution on pathology (yes, %)	80.8	100	.02
	Within JC by explant pathology (yes, %)	3.9	0.0	.32
	Pathological poorly differentiation	42.3	70.6	.07

Abbreviations: AFP, alpha‐fetoprotein; DCP, des‐gamma‐carboxy prothrombin; ELD, model for end‐stage liver disease; GRWR, graft recipient weight ratio; MR, lymphocyte‐to‐monocyte ratio; NLR, neutrophil‐to‐lymphocyte ratio; SLW, standard liver weight; W, graft weight.

**FIGURE 2:**
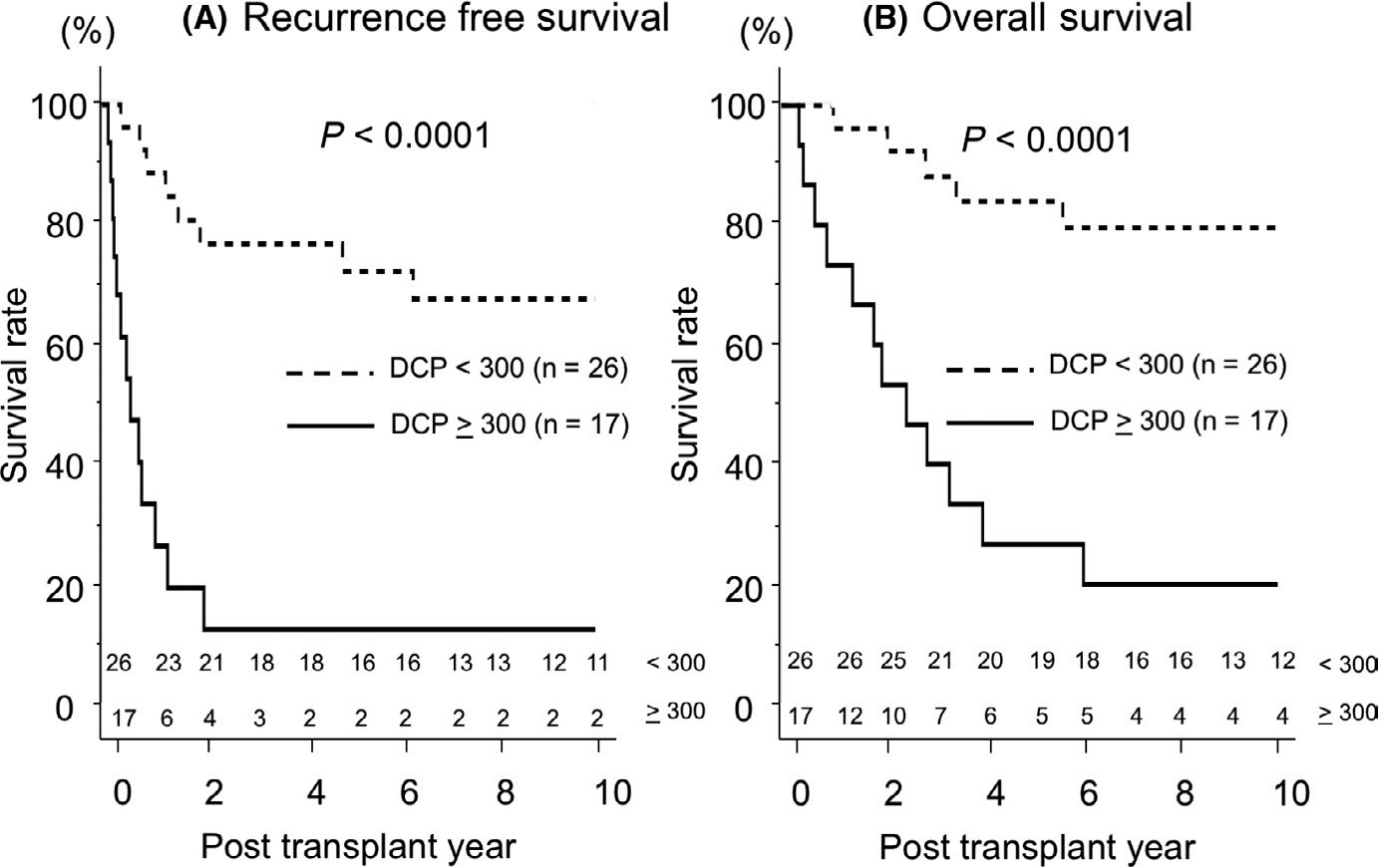
Recurrence‐free and overall survival after LDLT for HCC beyond the JC. A, Recurrence‐free survival of recipients beyond the JC divided according to DCP < 300 mAU/mL (n = 26) or DCP ≥ 300 mAU/mL or more (n = 17). B, Overall survival of recipients beyond the JC divided according to DCP < 300 mAU/mL (n = 26) or DCP ≥ 300 mAU/mL or more (n = 17)

Figure 2 shows the recurrence‐free and overall survival rates among patients beyond the JC. The patients were divided into two groups according to DCP levels. The 1‐, 5‐, and 10‐year recurrence‐free survival rates in patients with DCP < 300 mAU/mL were 88.3%, 71.5%, and 66.7%, respectively. The rates in patients with DCP ≥ 300 mAU/mL were 34.4%, 13.8%, and 13.8%, respectively (*P* < .0001, Figure 2A). The 1‐, 5‐, and 10‐year overall survival rates in patients with DCP < 300 mAU/mL were 100%, 83.2%, and 78.6%, respectively. In contrast, the rates in patients with DCP ≥ 300 mAU/mL were 73.3%, 26.7%, and 20.0%, respectively (*P* < .0001, Figure 2B).

## 4 DISCUSSION

Des‐gamma‐carboxy prothrombin was an independent risk factor for HCC recurrence and HCC‐related mortality after LDLT for patients beyond the JC. This study revealed that patients beyond the JC had a 65% of chance of surviving for more than 10 years after LDLT when their pre‐transplant DCP levels were <300 mAU/mL, even though Japanese insurance does not cover the medical cost for these cases. DCP level is well established as a sensitive and specific tumor marker in patients with HCC and is an independent predictive factor of microvascular invasion.^18^ We previously reported that DCP level was significantly correlated with macroscopic invasion and intrahepatic metastasis in the explanted liver,^11^ and was a predictor of microvascular invasion even for HCC of ≤3 cm.^19^ As shown in Table 4, patients with DCP ≥ 300 mAU/mL had significantly more microvascular invasion. DCP enhanced cell proliferation by Met receptor and angiogenesis by vascular endothelial growth factor.^19^ Patients beyond the JC with a DCP ≥ 300 mAU/mL had larger sized and more numerous tumors in bilateral lobes and higher AFP, which may reflect the ability of these tumors and the poorer prognosis. Furthermore, patients beyond the JC with a DCP ≥ 300 mAU/mL had higher NLR and lower LMR in this study.

Systemic inflammation is strongly associated with malignant tumor patient prognosis.^20^ Preoperative elevated NLR is an adverse predictor of recurrence‐free survival for patients undergoing hepatic resection for HCC,^21^ increases the risk of HCC recurrence after LT,^22^ and correlates with microvascular invasion and poorly differentiated tumors in explanted livers.^4^, ^23^ Infiltration of proinflammatory macrophages, cytokines, and chemokines in the tumor microenvironment boosted tumor growth, invasion, and metastases.^24^ Another study showed that interleukin (IL)‐17‐producing T cells released chemokines that recruited neutrophils leading to elevated NLR and promoted the differentiation of tissue macrophages in peritumoral regions into tumor‐associated macrophages (TAMs). Both IL‐17‐producing T cells and TAMs may accelerate tumor progression and antitumor T‐cell exhaustion.^22^ We recently reported the impact of LMR in resection or LDLT for HCC patients.^12^, ^21^ Lower LMR was an independent prognostic factors, particularly among patients beyond the MC after LDLT. LMR reflected the immune status of the tumor microenvironment in the explanted liver.^12^ This suggested that a low LMR indicated a greater number of macrophages and/or TAMs in the explanted liver.^12^ Furthermore, lower LMR was associated with high programmed death ligand‐1 expression in HCC.^21^ Modulation of the immune checkpoint pathway in the tumor microenvironment is associated with low LMR. Consequently, LMR ≤ 2.75 was an independent risk factor of HCC‐related mortality after LDLT in patients beyond the JC in this study.

In this study, DCP was measured immediately before transplantation because all patients underwent scheduled LDLT without a long waiting time. Halazun et al recently reported dynamic changes in AFP that reflected the treatment response after locoregional therapy, or as a surrogate for the biological behavior of patients, without treatment on a waiting list.^25^ They stated that many criteria use AFP at a single timepoint, even though patients usually wait a long time until LT and undergo locoregional therapy for HCC during the waiting period. Therefore, they hypothesized that the dynamic changes in AFP were a better predictor of recurrence and survival. As shown in Tables 1 and 4, >80% of the patients beyond the JC received pre‐transplant locoregional therapy; therefore, dynamic changes in DCP from the initial treatment to pre‐transplant might be a better predictor of the outcome of LDLT.

DCP ≥ 300 mAU/mL was an independent risk factor for HCC‐related mortality after LDLT in patients beyond the JC. Those patients had a higher MELD score and more blood loss compare with patients with DCP < 300 mAU/mL. A high MELD score^26^ or more blood loss usually reflects the poor status of a patient, which might affect the survival rate after LDLT. Indeed, among our 678 adult LDLT patients, those with MELD ≥ 22 (82.9% vs 92.2%, *P* = .0007) or blood loss > 20L (57.6% vs 92.0%, *P* < .0001) had poorer 6‐month patient survival rates after LDLT (data not shown). Causes of death among 15 lost patients with DCP ≥ 300 mAU/mL patients beyond the JC were HCC recurrence in 13 and graft failure in two. In contrast, 10 patients with DCP < 300 mAU/mL among 26 patients beyond the JC died after LDLT. Causes of death in these patients were HCC recurrence in five, HCV recurrence in two, pneumonia in two, and liver infarction in one. Consequently, HCC‐related mortality was used to analyze overall survival in this study.

Pre‐transplant ^18^F‐FDG positron emission tomography (PET) positivity is a prognostic factor for HCC recurrence or overall survival after LDLT.^27^ High standardized uptake values by PET usually reflects poorly differentiated HCC, combined HCC, or HCC with sarcomatous change. Although PET has a high cost and is not performed universally, it offers additional information when deciding on the indication of LDLT for HCC patients beyond the JC.

Limitations of the present study were its retrospective design and relatively small sample size, especially patients beyond the JC. A future multi‐institutional study is necessary although many candidates cannot be expected among the Japanese cohort. Because LDLT for patients within the MC has been covered by health insurance since 2004 in Japan, most patients undergoing LDLT are within the MC, even if a retrospective nationwide survey was performed.

In conclusion, the outcome of LDLT for patients with HCC within the JC was outstanding. The independent risk factor of HCC recurrence after LDLT for patients beyond the JC was DCP ≥ 300 mAU/mL. Independent risk factors for patient HCC‐related mortality were DCP ≥ 300 mAU/mL and LMR ≤ 2.75. Patients beyond the JC with DCP ≥ 300 mAU/mL might be contraindicated for LDLT.

## DISCLOSURES

Funding: This work was partly supported by JSPS KAKEN (grant numbers 15H0579 and 18K08542), and by the Program for Basic and Clinical Research on Hepatitis from the Japan Agency for Medical Research and Development (AMED 18fk0210023h0002).

Conflict of Interest: Authors declare no conflict of interests of this article.

Author Contributions: Yusuke Yonemura wrote the paper; Tomoharu Yoshizumi designed and performed the study, collected and analyzed the data; Shoichi Inokuchi, Yukiko Kosai‐Fujimoto, Noboru Harada, Shinji Itoh, Takeo Toshima, Kazuki Takeishi and Shohei Yoshiya performed the study and collected the data; Masaki Mori provided critical comments.

Ethical Statements: The study protocol was approved by the institutional review board (Approved number 2019‐186).
